# HER2 inhibitor-based combination therapy for recurrent and metastatic salivary duct carcinoma in an elderly patient: a case report and literature review

**DOI:** 10.3389/fonc.2025.1590497

**Published:** 2025-10-20

**Authors:** Luxin Zhang, Xiaofeng Xu, Liujie Gao, Jiyuan Ding

**Affiliations:** ^1^ Second Clinical Medical College, Zhejiang Chinese Medical University, Hangzhou, Zhejiang, China; ^2^ Department of Oncology and Hematology, Hangzhou Red Cross Hospital, Hangzhou, Zhejiang, China

**Keywords:** HER2, salivary duct carcinoma, submandibular gland, elderly patients, case report

## Abstract

Salivary duct carcinoma (SDC) is a rare and highly aggressive malignancy with limited treatment options, particularly in elderly patients. HER2 overexpression has emerged as a potential therapeutic target in this disease. This study reports the case of an 86-year-old male with HER2-positive submandibular gland SDC who underwent surgical resection on June 19, 2020. Six months postoperatively, follow-up revealed lymph node metastasis and local recurrence at the left submandibular region. Ultrasound-guided radiofrequency ablation was performed, but local recurrence persisted. The patient subsequently received trastuzumab combined with low-dose nab-paclitaxel, achieving a partial response according to RECIST 1.1 criteria. Maintenance therapy with trastuzumab monotherapy was then initiated, resulting in disease stability for over 20 months. In October 2023, the disease progressed to the left sublingual region. After targeted monotherapy and local radiotherapy by April the following year, disease control was achieved. At the most recent follow-up, the patient remains in stable condition. This case highlights the efficacy and safety of HER2-targeted combination therapy in elderly SDC patients, offering valuable insights into biomarker-driven personalized treatment strategies for this population.

## 1 Introduction

Salivary duct carcinoma (SDC) is one of the most aggressive subtypes of salivary gland carcinomas (SGCs), a rare group of malignancies accounting for less than 5% of all head and neck neoplasms (1). Clinically, SDC most frequently arises in the parotid gland, followed by the submandibular gland and minor salivary glands. It typically presents as a by rapidly enlarging neck masses, often accompanied by facial nerve paralysis and cervical lymph node metastasis. Owing to its highly invasive nature and propensity for early metastasis, SDC generally carries a poor prognosis, with a 5-year disease-specific survival rate of only 40-60%. Furthermore, the majority of cases are diagnosed at advanced stages, which further compromises treatment outcomes (2).

SDC has an aggressive clinical course and poor prognosis, making therapeutic strategies challenging, especially in elderly patients with reduced tolerance to conventional treatments. Molecular targeted therapies, particularly HER2 inhibitors, have emerged as a promising approach. However, evidence for their use in elderly populations remains limited (3). This study presents a case report of an 86-year-old man with HER2-positive submandibular gland SDC who underwent HER2 inhibitor-based combination therapy following postoperative recurrence. Follow-up evaluations revealed sustained disease stability over four years with treatment-related toxicity remaining within manageable limits. This case highlights the efficacy and safety of HER2-targeted combination therapy in elderly SDC patients, offering valuable insights into biomarker-driven personalized treatment strategies for this population.

## 2 Case report

An 86-year-old man patient was admitted to our hospital on June 17, 2020, presenting with a ten-year history of a left submandibular mass and newly developed left-sided lingual numbness and paresthesia persisting for one week. The patient had been previously asymptomatic and had not sought medical attention for the mass until the recent onset of symptoms. On physical examination a firm, approximately 3.5 cm mass was palpated in the left submandibular region, exhibiting fixation to the overlying skin and restricted mobility. The tongue was midline at rest with preserved mobility.

**Figure 1:**
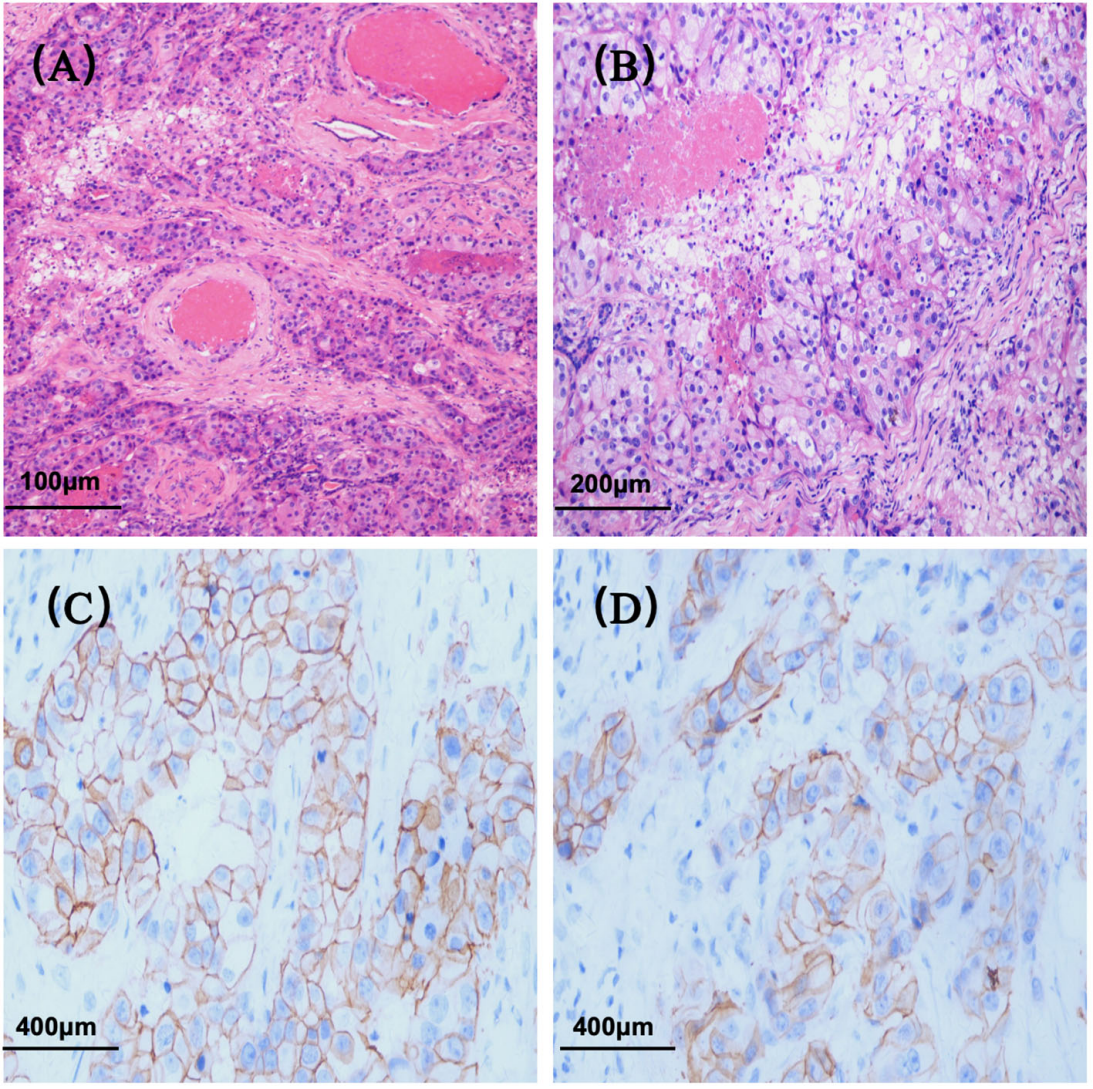
Histopathological features of salivary duct carcinoma. **(A)** Hematoxylin and eosin (H&E) staining (original magnification ×100); **(B)** H&E staining (original magnification ×200); **(C)** Immunohistochemical staining for HER2 (original magnification ×400); **(D)** HER2 immunohistochemistry (original magnification ×400).

Initial diagnostic imaging included magnetic resonance imaging (MRI) of the head and neck, which identified a 1.5×1.3×1.1 cm calcified lesion within the left submandibular gland (**Figure 1**). Subsequent contrast-enhanced ultrasonography revealed a heterogeneous hypoechoic nodule with prominent internal vascularity in the left submandibular gland. Fine-needle aspiration biopsy (FNAB) confirmed the presence of infiltrating atypical glandular tissue, suggestive of submandibular gland malignancy.

**Figure 2:**
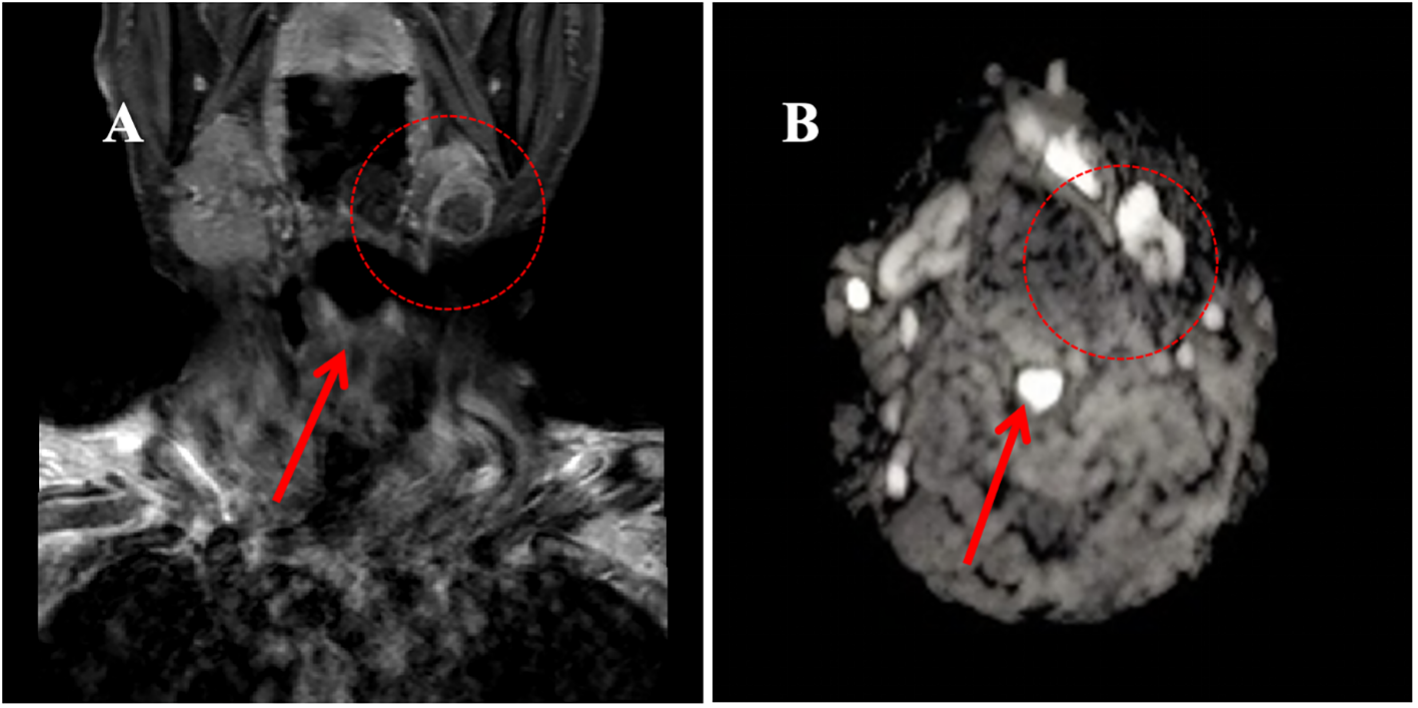
Magnetic Resonance Imaging (MRI) of the neck region in June 2020. **(A)** T1-weighted image showing a hypointense lesion in the left submandibular gland (approximately 1.5×1.3×1.1 cm); **(B)** Diffusion-weighted image demonstrating a hyperintense signal in the left submandibular gland.

The patient underwent left submandibular gland excision on June 19, 2020, for definitive diagnosis. Histopathological examination confirmed ductal carcinoma (pT2NxM0) with perineural and vascular invasion. Immunohistochemical analysis demonstrated positive staining for CK7, AR, and HER2 (3+) (**Figure 2**), with strong p53 expression (+++) and a Ki-67 proliferation index of 35%. Negative markers included CK5/6, GCDFP-15, SMA, p40, p63, CD117, and Calponin (**Table 1**). The patient was followed up regularly without any adjuvant therapy.

**Table 1:** Immunohistochemical Staining Control.

Immunohistochemical staining	Jun-20	Oct-21
Positive marker		
CK7	(+)	
AR	(+)	(+)
HER2	(3+)	(3+)
p53	(+++)	
Ki-67	35%	
GCDFP-15	(-)	(+)
CAM5.2		(+)
MSH2		(+)
MSH6		(+)
PMS2		(+)
MLH1		(+)
Negative Marker		
CK5/6	(-)	
SMA	(-)	
p40	(-)	(-)
p63	(-)	(-)
CD117	(-)	
Calponin	(-)	
PSA		(-)
TTF-1		(-)
NapsinA		(-)
CK20		(-)
CDX2		(-)
PD-1		(-)
ER		(-)

Blank spaces indicate items not assessed in the current examination.

By April 2021, the patient developed progressive lingual sensory disturbance, dysarthria, and leftward tongue deviation. Cervical ultrasonography revealed lymph node metastasis and local recurrence at the left submandibular region, confirmed by biopsy. The recurrent lesion was then treated with ultrasound-guided radiofrequency ablation.

**Figure 3:**
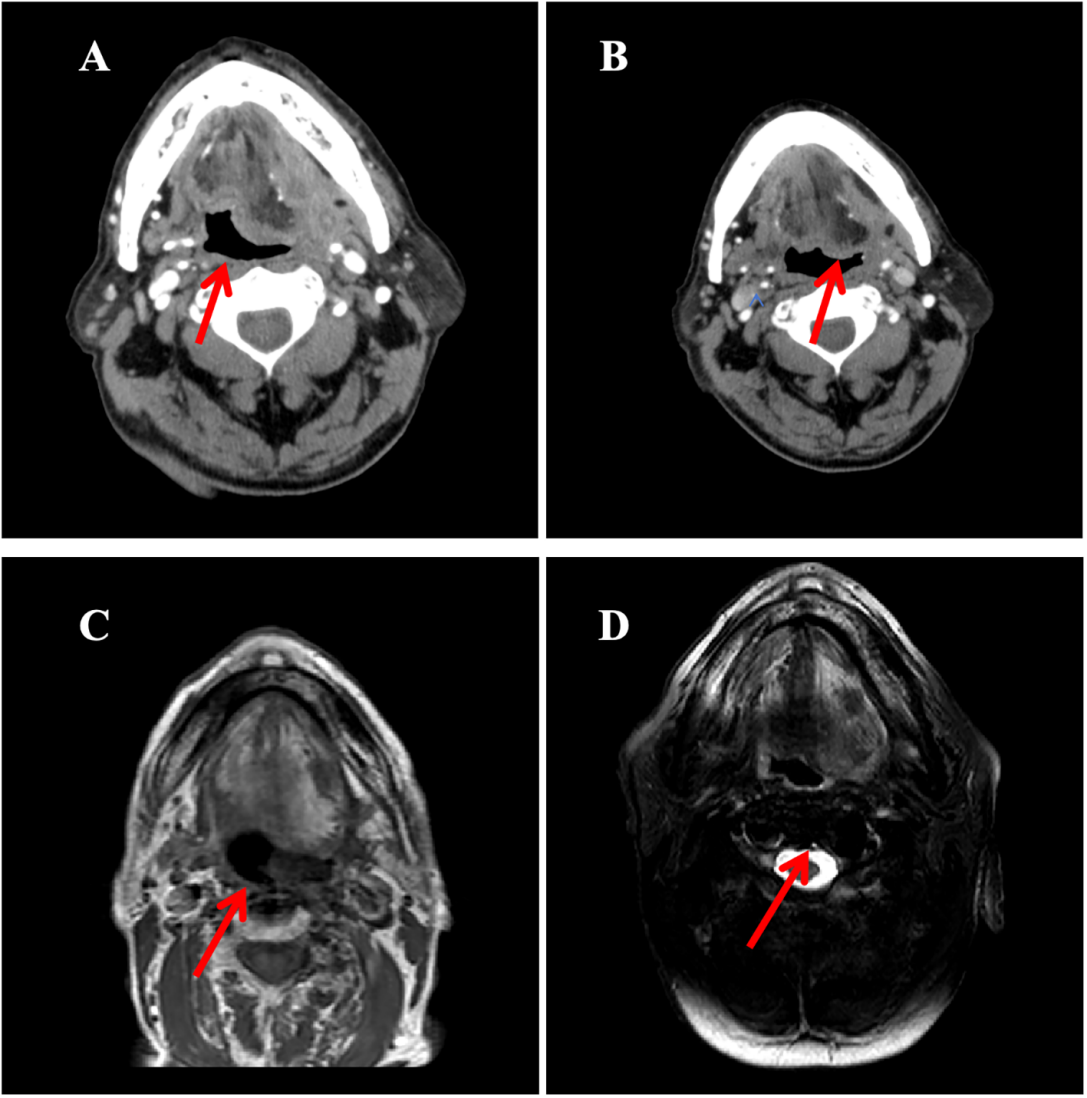
The imaging evolution of a left submandibular mass in a patient during the treatment period from November 2021 to October 2023. **(A)** Contrast-enhanced neck CT (October 2021): Demonstrates an enhancing mass in the left submandibular region (maximum diameter: 5.0 cm); **(B)** Contrast-enhanced neck CT (February 2022, After 4 cycles of treatment): Shows significant reduction in the size of the mass compared to A (maximum diameter: 3.2 cm); **(C)** Contrast-enhanced MRI of the submandibular gland (axial T1-weighted image; March 2023): Reveals a mass in the sublingual region (maximum diameter: 1.7 cm); **(D)** MRI of the submandibular gland (axial T1-weighted image; June 2023): The mass measures 1.8 cm in maximum diameter, indicating size stability compared to C.

In October 2021, the patient experienced progressive worsening of symptoms and was subsequently transferred to our department for further management. Contrast-enhanced CT of the tongue demonstrated an irregular soft tissue density lesion (2.1×5.0 cm) in the left submandibular region with heterogeneous enhancement (**Figure 3**). Biopsy confirmed poorly differentiated invasive carcinoma with immunohistochemical profile consistent with the primary lesion (**Table 1**), confirming SDC origin.

Based on HER2 overexpression, advanced age, and poor performance status, the patient started trastuzumab (loading dose 8 mg/kg, maintenance dose 6 mg/kg q3w) combined with nab-paclitaxel (80 mg/m² on days 1 and 8, q3w) in November 2021. After four cycles, contrast-enhanced CT scan of the tongue showed a partial response (PR), with a reduction in the size of the submandibular lesion. Two additional cycles were administered as consolidation therapy, achieving radiological stability. Maintenance therapy with trastuzumab (6 mg/kg q3w) began in April 2022, with imaging follow-up scheduled every 12 weeks (every 4 treatment cycles). However, due to poor patient compliance, only four cycles were intermittently completed between April 2022 and June 2023.Imaging evaluations during this period confirmed stable disease (SD) according to RECIST 1.1 criteria (**Figure 3**).

In October 2023, follow-up contrast-enhanced MRI of the head and neck revealed a lesion in the left sublingual region. Combined with clinical findings, this was assessed as disease progression according to RECIST 1.1 criteria. Targeted monotherapy was recommended but the patient did not adhere to the treatment regimen.

By March 2024, enhanced MRI of the mandible showed enlargement of the left sublingual mass (maximum diameter 4.1 cm) accompanied by progressive worsening of symptoms. Following comprehensive assessment, radiotherapy to the submandibular region was initiated on 9th April 2024 (total dose 60 Gy/30 fractions, 2 Gy/fraction), resulting in symptom relief. The patient remained in stable condition at the most recent follow-up in January 2025.

## 3 Discussion

SDC is a rare, highly aggressive malignancy characterized by high rates of local recurrence, poor prognosis, and significant mortality (4).Standard treatment involves complete surgical resection followed by postoperative radiotherapy (5). For inoperable, recurrent, or metastatic disease, chemotherapy is the mainstay, though no standardized regimen exists. Given the limitations of single-modality treatments, a multimodal approach is often optimal.

In recent years, molecular profiling has gained increasing importance in SDC, and HER2-targeted therapy has attracted growing interest. Several international guidelines now recommend HER2 status assessment in the diagnostic workup of recurrent or metastatic SDC (6). This approach is supported by the notable morphological and molecular similarities between SDC and high-grade breast ductal carcinoma. Trastuzumab, a humanized monoclonal antibody targeting the extracellular domain of HER2, inhibits downstream signaling and suppresses proliferation of HER2-overexpressing tumor cells (7). Its efficacy, well-established in breast cancer through large-scale randomized trials, provides a rationale for its application in SDC.

Building on the success in breast cancer therapy and supported by clinical evidence in SDC, several phase II trials have evaluated the efficacy of trastuzumab-based targeted therapy combined with chemotherapy in SDC, demonstrating promising outcomes with objective response rates (ORR) ranging from 58% to 70% and median progression-free survival (MPFS) ranging from 6.9 to 11.7 months (8–11). Notably, this approach has shown promising clinical efficacy even in patients with recurrent or metastatic disease, For example, a recent phase II clinical trial conducted by Lee et al. reported a 70% ORR for trastuzumab combined with taxane therapy in metastatic SDC, which is consistent with the partial response observed in our case. Notably, while the combination of trastuzumab and docetaxel has become a recommended regimen for salivary duct carcinoma (SDC), it is associated with a high incidence (approximately 50%) of grade ≥3 treatment-related adverse events (12). Data in frail elderly patients are scarce. Our use of low-dose nab-paclitaxel aimed to optimize tolerability.

This trastuzumab-based, low-dose nab-paclitaxel regimen represents a deviation from standard therapy, aiming to balance efficacy and safety. In our case, the clinical outcome was consistent with previous studies. Despite suboptimal treatment compliance during the maintenance phase due to the patient’s frail condition, local disease control was sustained for over 20 months. This suggests that a HER2-targeted, low-intensity chemotherapy combination can yield durable efficacy even in elderly, vulnerable patients, though its definitive value requires confirmation in prospective studies. Therefore, comprehensive molecular testing is recommended for this population.

The optimal duration of HER2 inhibitor maintenance therapy remains undefined. In numerous cases of SDC treatment, the duration of targeted maintenance therapy varies significantly, with some patients achieving favorable outcomes after only one year of trastuzumab maintenance therapy (13, 14). In our case, disease progression occurred after one year of maintenance therapy, which may be related to irregular treatment adherence. The outcome appears slightly inferior compared to other reported cases, suggesting that maintenance therapy should be stratified according to disease stage. However, specific protocols require further investigation through larger sample sizes. Ultimately, treatment decisions should be based on comprehensive assessment of patient status and treatment goals, incorporating the best available evidence.

Adjuvant radiotherapy has demonstrated significant efficacy in the management of head and neck cancers. Current evidence suggests substantial clinical benefits of postoperative radiotherapy in SDC patients, particularly those with high-risk features such as extracapsular extension and/or positive margins. This approach has been shown to improve overall survival and enhance locoregional control. And there are Studies have consistently identified the omission of postoperative radiotherapy as a significant risk factor for disease recurrence (15).

Unfortunately, the patient did not receive standard postoperative radiotherapy initially, due to comorbid conditions and patient preference. Subsequently, the patient developed lymph node metastasis within six months after surgery, suggesting that the omission of adjuvant radiotherapy may have compromised disease control.

It is worth noting that radiotherapy may still play an important role within multimodal therapy even in recurrent or metastatic settings. For example, Rencui Qua et al. reported a case of SDC with postoperative lymph node metastasis treated with trastuzumab, chemotherapy, and concurrent regional radiotherapy, achieving a complete response lasting five years (16). Had our patient received concurrent radiotherapy during HER2-targeted treatment—if medically feasible—it might have provided additional benefit, potentially improving disease control and prognosis.

Other therapeutic avenues for recurrent/metastatic SDC include androgen deprivation therapy (ADT) and immunotherapy (17, 18). Approximately 70% of SDCs express androgen receptor (AR), and ADT is gaining traction due to its favorable toxicity profile and efficacy (19). Although our patient was AR-positive, ADT was not initially considered due to limitations in clinical consensus and therapeutic depth at the time. Current evidence suggests that targeted therapy should take precedence, but combination with ADT may prolong survival (20). For selected patients, adjuvant ADT or ADT-based combination therapy may represent a promising strategy, further refining personalized treatment approaches.

In conclusion, SDC is a rare and highly aggressive malignancy with significant therapeutic challenges, particularly in elderly patients. Identifying unique biomarkers, such as HER2, and implementing personalized treatment strategies are crucial. HER2 inhibitor-based combination therapy has shown efficacy and manageable toxicity in elderly SDC patients with local recurrence and metastasis. However, further clinical studies are needed to refine and optimize treatment protocols.

## Data Availability

The raw data supporting the conclusions of this article will be made available by the authors, without undue reservation.
